# Phase Transformation in 316L Austenitic Steel Induced by Fracture at Cryogenic Temperatures: Experiment and Modelling

**DOI:** 10.3390/ma14010127

**Published:** 2020-12-30

**Authors:** Kinga Nalepka, Błażej Skoczeń, Marlena Ciepielowska, Rafał Schmidt, Jakub Tabin, Elwira Schmidt, Weronika Zwolińska-Faryj, Robert Chulist

**Affiliations:** 1 Faculty of Mechanical Engineering and Robotics, AGH University of Science and Technology, 30-059 Krakow, Poland; zwolinska@agh.edu.pl; 2 Faculty of Mechanical Engineering, Cracow University of Technology, 31-155 Krakow, Poland; bs@mech.pk.edu.pl (B.S.); marlena.ciepielowska@doktorant.pk.edu.pl (M.C.); rafal.schmidt@pk.edu.pl (R.S.); elwira.schmidt@doktorant.pk.edu.pl (E.S.); 3 Institute of Fundamental Technological Research, 02-106 Warsaw, Poland; jtabin@ippt.pan.pl; 4 Institute of Metallurgy and Materials Science, Polish Academy of Sciences, 30-059 Krakow, Poland; r.chulist@imim.pl

**Keywords:** austenitic steel, cryogenic temperatures, fracture process, fcc-bcc phase transformation, synchrotron radiation, electron backscatter diffraction, XFEM simulation

## Abstract

Investigations by electron backscatter diffraction (EBSD) and X-ray diffraction with the use of synchrotron radiation, as well as parallel extended finite element (XFEM) simulations, reveal the evolution of the 316L stainless steel microstructure in the vicinity of a macro-crack developing at the temperature of liquid helium (4.2 K). The fracture propagation induces a dynamic, highly localized phase transformation of face-centred cubic austenite into α’ martensite with a body-centred cubic structure. Synchrotron studies show that the texture of the primary phase controls the transition process. The austenite grains, tending to the stable Brass orientation, generate three mechanisms of the phase transformation. EBSD studies reveal that the secondary phase particles match the ordered austenitic matrix. Hence, interphase boundaries with the Pitsch disorientation are most often formed and α’ martensite undergoes intensive twinning. The XFEM simulations, based on the experimentally determined kinetics of the phase transformation and on the relevant constitutive relationships, reveal that the macro-crack propagates mainly in the martensitic phase. Synchrotron and EBSD studies confirm the almost 100% content of the secondary phase at the fracture surface. Moreover, they indicate that the boundaries formed then are largely random. As a result, the primary beneficial role of martensite as reinforcing particles is eliminated.

## 1. Introduction

The dynamic development of science and technology means that more and more devices work in the range of extremely low temperatures of liquid nitrogen (77 K), liquid helium (4.2 K), or even superfluid helium (below 2.17 K). Excellent examples are the superconductor-based applications: nuclear magnetic resonance, particle accelerators and energy transmission grids. Conditions of cryogenic temperature also prevail in the cosmic space. Hence, they are crucial for the construction of orbiters, probes, and shuttles. The failure-free operation of these highly specialized devices and machines requires the use of appropriate material. The most commonly applied are stainless steels of the 304, 316 or 321 grades, which deform plastically practically down to absolute zero [1,2,3]. Even if their physical and mechanical properties considerably evolve, they retain high ductility reaching the rupture strain of about 40–50%. This is fundamental for very demanding service conditions. The reduction of temperature to the proximity of absolute zero induces a considerable increase in the yield stress, which is related to the transformation of the face-centred cubic (fcc) austenite into the body-centred cubic (bcc) α’ martensite [4]. As a result, the initial material changes into a two-phase continuum composed of the plastic matrix and the secondarily arising hard inclusions [5]. At cryogenic temperatures, in addition to the direct transition γ → α’, there is a three-stage transformation γ→ε→α’. Thus, apart from austenite and α’ martensite, an intermediate phase—ε martensite with a hexagonal close packed structure (hcp) is locally present. It constitutes a product of incomplete transformation.

One of the key issues remains the initiation of the martensitic transformation. This problem has been studied extensively in the temperature range of liquid nitrogen and above. Then, formation of the new phase requires the yield point to be reached. The so-called plastic strain-induced transformation takes place. The basic mechanism of nucleation is shear in the intersecting planes of close packing [6,7]. The displacements necessary for the transformation of the fcc structure into bcc are parts of the twinning shear of austenite *T*: *T*/3 and 3*T*/8, respectively. Thus, the shears $\frac{a}{18}\left[1\;\bar{2}\;\bar{1}\right]{\;}_{\gamma }$ on $\left(1\;1\;\bar{1}\right){\;}_{\gamma }$ and $\frac{a}{16}\left[1\;\bar{2}\;1\right]{\;}_{\gamma }$ on $\left(1\;1\;1\right){\;}_{\gamma }$ generate the martensitic nucleus with the habit $\left(1\;1\;1\right){\;}_{\gamma }$. The liquid helium temperature requires a separate approach. Close to absolute zero, the stresses become an important factor influencing the initiation and course of the phase transition. Moreover, plastic deformation occurs mainly through discontinuous flow, which is coupled with the transformation γ → α’. Due to the complex behaviour of the material, the mechanisms of martensite nucleation and its microstructure have not been thoroughly investigated so far. This issue has been considered in few papers [8,9].

The situation is different in the case of martensite nucleation kinetics, which does not require in-depth analysis of the microstructure. Extensive research on the subject was initiated by Olson and Cohen [10]. The physically based model, including 3 parameters, was calibrated against the curve reflecting the amount of martensite versus plastic strain. Narutani, Olson and Cohen [11] developed a more advanced model that predicts the behaviour of metastable austenite at extremely low temperatures. The flow stress was controlled by two factors: static hardening and dynamic softening. Stringfellow, Parks, and Olson [12] generalized the Olson-Cohen kinetics, including the evolution of the secondary phase depending on temperature, plastic strain and stress. In their model, the nucleation strain was composed of deviatoric and hydrostatic terms. In addition, the isotropic hypo-elastic approach was applied. The Eshelby model, including spherical and isotropic inclusions implanted in an infinite and incompressible matrix, was used. Plastic flow observed during the phase transformation process has been modelled by Leblond et al. [13] for such cases when the Magee mechanism was negligible. In the model, the phases were assumed to be perfectly plastic. Later on, the model was extended to a mixed (isotropic and kinematic) hardening version. Fischer et al. [14] developed a micro-mechanical constitutive model, including coupling between micro-plasticity and phase transformation. Assessment of the volume fraction of martensite for low stacking fault energy (LSFE) steels, subjected to thermo-mechanical loads, was carried out by Diani and Parks [15]. The formation of shear-bands locked by the secondary phase was analysed. At extremely low temperatures, two mechanisms of shear-band formation were observed: the formation of ε-martensite and twinning. In their micro-mechanical model, the authors assumed no more than two active slip systems for each grain. Another thermo-mechanical approach to the plastic strain induced fcc-bcc phase transformation was developed by Levitas, Idesman and Olson [16]. Finite inelastic and small elastic strains were accounted for. The deformation gradient was decomposed into elastic, transformation and plastic parts. The generalized Prandtl-Reuss model was applied. In a new constitutive approach, Fischer et al. [17] took into account the effect of transformation induced plasticity (TRIP) and shear on the irreversible deformation. Another micro-mechanical analysis was developed by Cherkaoui et al. [18]. The internal stress sources related to the incompatible transformation strain or to the plastic flow of both phases were analysed. The micro domains of the secondary phase were represented by the ellipsoidal type Eshelby inclusions with a specific aspect ratio. The transition from single to polycrystal was carried out by means of a self-consistent algorithm.

In view of the above, the aim of the present work is to identify the evolution of the 316L stainless steel microstructure in the vicinity of a macro-crack developing at the temperature of liquid helium (4.2 K). The obtained results are presented in the context of liquid nitrogen and room temperatures. In-depth analysis of the microstructural changes was carried out by combining two techniques: X-ray diffraction using synchrotron radiation and the diffraction of backscattered electrons (EBSD). Examination of samples with symmetric notches subjected to the tensile test reveals the texture of the primary phase and the distribution of the secondary phase at different distances from the fracture surface. Including EBSD investigations enables the identification of martensite nucleation mechanisms closely related to the preferred austenite orientation. In order to obtain the complete characteristics of the microstructure, the disorientations at the interphase boundaries and the relationships between the martensite grains are also determined. It turns out that they are not random. The second part of the research includes numerical modelling of the phase transformation associated with the macro-crack propagation. The extended finite element method (XFEM) is used and the simulations are based on the experimentally determined martensite nucleation kinetics and on the relevant constitutive relations. When identifying the phase transformation kinetics, the linear relationship between the rates of martensite formation and the plastic strain was adopted. The combination of numerical analysis with earlier identification of the microstructure allows the conditions under which the fracture develops to be revealed.

## 2. Materials and Methods

**Figure 1:**
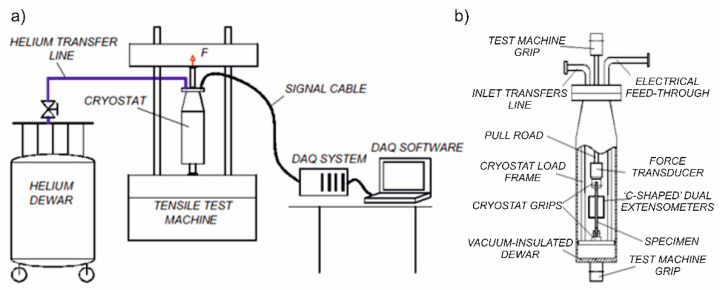
(**a**) Experimental set-up for a uniaxial tensile test at cryogenic temperatures; (**b**) cryostat with the specimen and transducers.

The set-up for cryogenic testing comprises a number of components mounted in the dedicated traction machine [3,19]. In particular, a cryostat containing the specimen as well as the strain transducers were installed between the grips of the testing machine. The cryogen (LHe or LN_2_) was poured into the cryostat by means of a dedicated flexible transfer line, composed of a corrugated stainless-steel hose. As long as the specimen with the transducers was not entirely immersed in the cryogenic medium, the test was on hold. The cryogen level was checked by means of a thermistor located inside the cryostat. The tests were carried out with the crosshead velocity of 1.0 mm/min. The results were recorded with the sampling rate of 1000 Hz. Such a sampling rate was selected in order to register the discontinuous plastic flow (DPF), which accompanies the plastic strain induced phase transformation in liquid helium [3,20,21]. Figure 1 illustrates the experimental set-up, as well as the cryostat interior.

**Figure 2:**
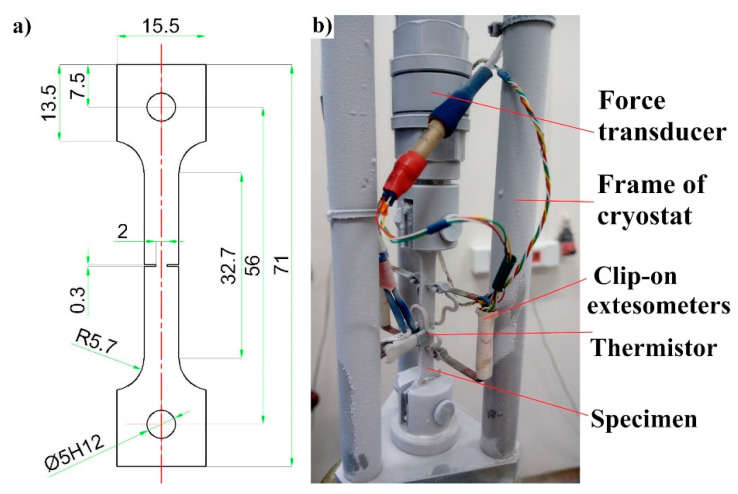
(**a**) Specimen geometry (thickness of 2 mm); (**b**) the cryostat arrangement- specimen with transducers ready for the test at 4.2 K.

In order to trace the course of the fracture process, flat specimens with symmetric notches were used (Figure 2a). They were cut off from steel sheets by means of the WEDM (wire electrical discharge machining) method. perpendicularly to the rolling direction. The chemical composition of the specimens is listed in Table 1.

The complex set-up contains a double-wall vacuum insulated cryostat that is not transparent and does not allow the specimen to be observed directly. For this reason, a rather sophisticated set of instruments connected to a high-resolution data acquisition system (DAQ) has been used (Figure 2b). The specimen elongation was measured by using two clip-on extensometers [22], mounted on either side of the uniform gauge section in such a way that the notches were localized symmetrically between the arms of the extensometers. A piezoelectric force transducer was aligned with the specimen inside the cryostat in order to measure the force directly on the sample. The thermistor, installed in the central part of the gauge length, measured the temperature of the specimen surface. Referential measurements of the load were carried out using the load cell integrated in the traction machine, outside the cryostat. The sampling frequency is high enough to measure the abrupt drop of stress and increase in temperature during the serrations. The time delay for each transducer was far below the measurement period (1 ms). The crosshead velocity enabled observation of the DPF during the plastic deformation at cryogenic temperatures.

After the tests, selected parts of broken specimens were subjected to in-depth microstructural investigations. For this purpose, two techniques were applied: X-ray diffraction with the use of synchrotron radiation and electron backscatter diffraction (EBSD) employing an FEI Quanta 3D Scanning Electron Microscope (Hillsboro, Oregon, OR, USA). The first enabled identification of the evolution of phases and the texture of plastic austenite, while the other allowed mechanisms of the phase transformation to be revealed. The calculations were performed with the use of MTEX software [23].

The formation of martensite is related to local changes in austenite orientation. They induce lattice curvature, which is accommodated by so-called geometrically necessary dislocations (GND). In order to thoroughly examine the phase transformation, distributions of GND density were determined for the representative regions of the specimen stretched at the temperature of liquid helium. The approach proposed by Pantleon was used [24]. According to it, at points of the studied area, the curvature tensors $\kappa_{ij}\;\left(i,\;j=1,2,3\right)$ are identified. These characteristics are calculated on the basis of orientation differences registered in the EBSD image. Omitting the elastic strains, the quantity $\kappa_{ij}$ can be expressed by the total dislocation density tensor $\alpha_{ij}$ according to the Nye relationship $\kappa_{ij}=\alpha_{ji}-0.5\delta_{ij}\alpha_{mm}$ [25]. The lattice curvature at the measuring point induces various types of dislocations. Each of them is characterized by the appropriate Burgers vector $b^{t}$ and the line vector $l^{t}$, as well as the density $\rho^{t}$. Using the total dislocation density tensor expressed in the specimen system $\alpha_{ij}=\overset{N}{\underset{t}{\sum }}b_{i}^{s.t}l_{j}^{s.t}\rho^{t}$, one obtains the relationship between the sought dislocations and the measured elements of the curvature tensor $\kappa_{ij}^{s}=\overset{N}{\underset{t}{\sum }}\left(b_{j}^{s.t}l_{i}^{s.t}-0.5\delta_{ij}b_{m}^{s.t}l_{m}^{s.t}\right)\rho^{t}$. The EBSD study is performed in two-dimensional space. Thus, three elements $\kappa_{i3}^{s}$ remain unrecognised. As a result, six linear equations are obtained while the number of unknowns is significantly higher. A physically justified approach to solving this problem is minimizing the energy of the dislocations being identified. According to active slip systems, six Burgers vectors 〈1 1 0〉a/2  are taken into account in the calculations. They generate 6 screw and 12 edge dislocations. The double number of the latter results from the fact that two planes {1 1 1} intersect along one Burgers vector, which leads to two dislocation lines 〈1 1 2〉.

The phase analysis was performed using a synchrotron source at DESY, Hamburg, Germany employing a beam line P07 (λ = 0.0142342 nm). A beam with a cross-section of 0.7 × 0.7 mm^2^ was applied in five areas spaced equally 1 mm apart. The high energy of synchrotron radiation allowed the data to be recorded using transmission geometry, which significantly improved the statistical analysis. Additionally, to eliminate the texture effect, samples were continuously rotated during the measurement [26,27]. In order to calculate the volume fraction of particular phases, the Rietveld refinement using the FullProof Suite software (April2018, CEA, Saclay, France) was applied. Pole figures were computed with the use of the StressTexCalculator software [28]. The volume fractions of texture components are given with a spread of ±15°.

## 3. Experimental Results

### 3.1. Mechanical Response at Different Temperatures

**Figure 3:**
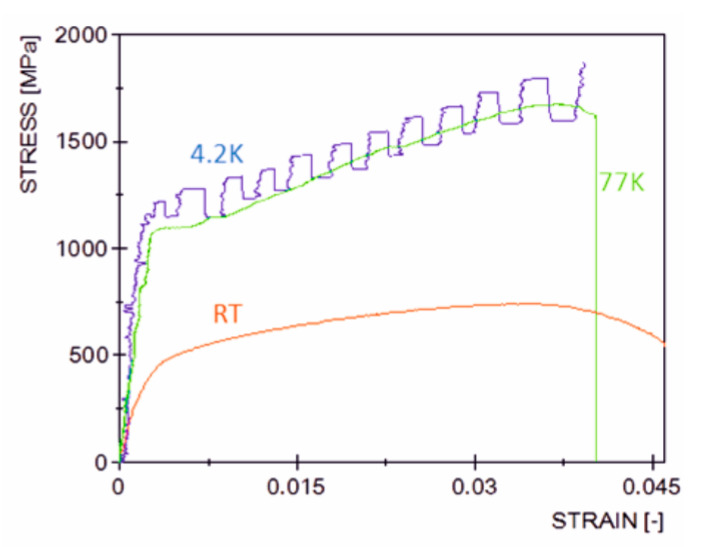
Stress versus strain for flat specimens with symmetrical notches.

Behaviour of the material during the axial tension at three different temperatures is presented in Figure 3, where the average engineering stress against the average Cauchy strain is depicted. The mechanical response of the material is smooth at room temperature and at 77 K. This indicates the classical mechanism of plastic flow, based on the massive motion of dislocations in the lattice. The initial part of plastic flow in liquid nitrogen (77 K) is nearly perfect, and the hardening occurs as soon as the phase transformation threshold is reached. It begins at small average strain (around 0.005), due to the strong strain localization and highly non-linear strain distribution in the vicinity of the notches. The material response in liquid helium (4.2 K) is completely different due to the occurrence of discontinuous plastic flow and strong stress-strain oscillations, indicating the oscillatory mode of plastic flow. This is mainly due to the mechanism of accumulation of dislocations in the form of pile-ups on the internal lattice barriers (weakly excited lattice), as well as the possible influence of twinning [29]. It is presumed that, as soon as the shear stress at the head of the dislocation pile-up reaches the level of the cohesive strength, the lattice barriers are overcome (broken) and the resulting massive release of dislocations is accompanied by the drop of stress. This phenomenon is not accounted for in the present paper, and the stress oscillations are treated as a secondary effect when compared to the fcc-bcc phase transformation, which remains the main focus of the paper. Hardening at 4.2 K begins roughly at the same level of strain as in the case of liquid nitrogen (77 K), and the hardening modulus is quite similar, which indicates a similar hardening mechanism. It is therefore assumed that, in both cases (4.2 K, 77 K), the hardening mechanism is based on the plastic strain induced fcc-bcc phase transformation.

### 3.2. Phase Evolution

**Figure 4:**
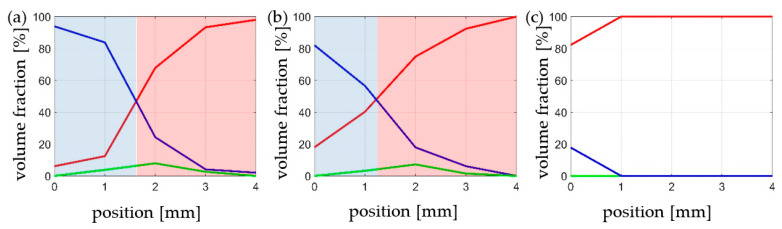
Volume fraction of austenite (red line), α’ martensite (blue line), and ε martensite (green line) as a function of a distance from the fracture surface for the specimens stretched at different temperatures: (**a**) 4.2 K, (**b**) 77 K, and (**c**) room temperature.

The stress concentration induced by symmetrical notches makes the distribution of α’ martensite to exhibit a very strong gradient. This was revealed by X-ray diffraction studies using synchrotron radiation (Figure 4). The measurements were performed in five areas spaced equally 1 mm apart. At the fracture surface of the specimen stretched at 4.2 K, the volume fraction of α’ martensite reaches 94%. Moving away from the fracture surface, the content of the hard α’ phase decreases. At a distance of less than 2 mm, it is the same as the austenite fraction, and at a distance of 4 mm, i.e., twice the width of the specimen at the site of notches, almost only austenite occurs (94% volume). Traction at 77 K induces a similar material behaviour. However, the volume content of α’ martensite at the fracture surface amounts to 82% and the decrease with distance is stronger. Synchrotron studies show that the areas of equal content of austenite and α’ martensite are correlated with the area of the highest fraction of transition phase—ε martensite. At room temperature (RT), α’ martensite is formed only at the fracture surface, and its volume fraction is 17.8%.

During the synchrotron investigations, the crystalline structure of individual phases was also identified. The lattice constants of fcc austenite and bcc α’ martensite are equal to 3.591 Å and 2.869 Å, respectively. Additionally, the hcp structure of ε martensite is characterized by the following parameters $a_{m\varepsilon }=2.191\;Å,\;c_{m\varepsilon }=4.110\;Å$.

### 3.3. Austenite Texture

**Figure 5:**
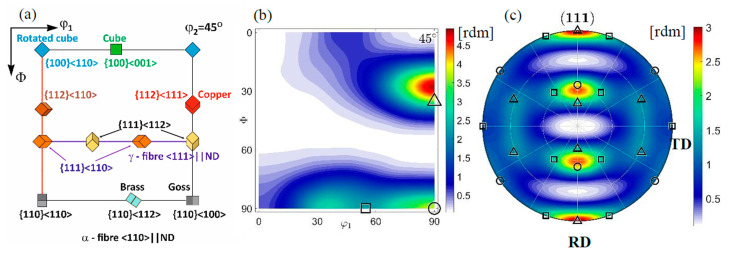
Initial texture: (**a**) key components and fibres in the section of Euler angle space at $\varphi_{2}$ = 45° (**b**) ODF with Brass (square), Goss (circle), Copper (triangle) orientations. The function values constitute multiples of a random misorientation (rdm). (**c**) {111} pole figure. ND, TD, and RD denote normal, transverse and rolling directions, respectively.

The initial texture, identified before tension, is similar to others observed in recrystallized cold rolled 316L steel [30,31]. Because of the difference in chemical composition, instead of the Brass dominant orientation, the Cu component prevails (Figure 5). Thus, the initial texture consists of the following components: Cu {1 1 2}<1 1 1>(16.2%), Goss {1 1 0}<0 0 1> (13.7%), and Brass {1 1 0}<1 1 2> (10.5%). The values were obtained on the basis of the orientation distribution function (ODF) determined by Labotex and StressTexCalculator software [27,32].

After the tension, independently of the temperature, the texture is quantitatively similar, while the intensities of individual components change. In the area of relatively small stresses located 4 mm from the fracture surface, the arrangement is mainly introduced by rolling. As a result, a texture consisting of the Brass {1 1 0}<1 1 2>, Goss {1 1 0}<0 0 1> and Cu {1 1 2}<1 1 1> components with slightly different contributions compared to the initial one is observed (see Table 2).

**Figure 6:**
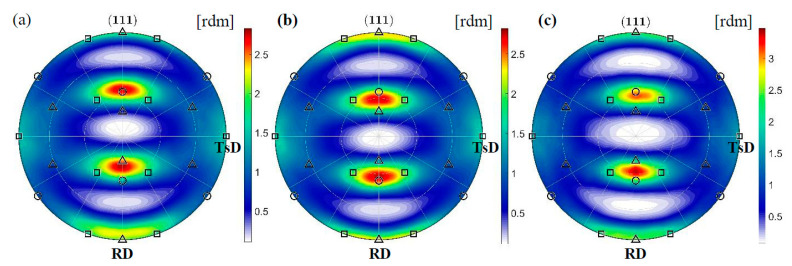
{1 1 1} pole figures for austenite in the area located 4 mm from the fracture surface of specimens stretched at the temperature of 4.2 K (**a**), 77 K (**b**) and RT (**c**). The Brass, Goss and Cu texture components are denoted by squares, circles and tringles, respectively. TsD || TD denotes the tensile direction.

**Figure 7:**
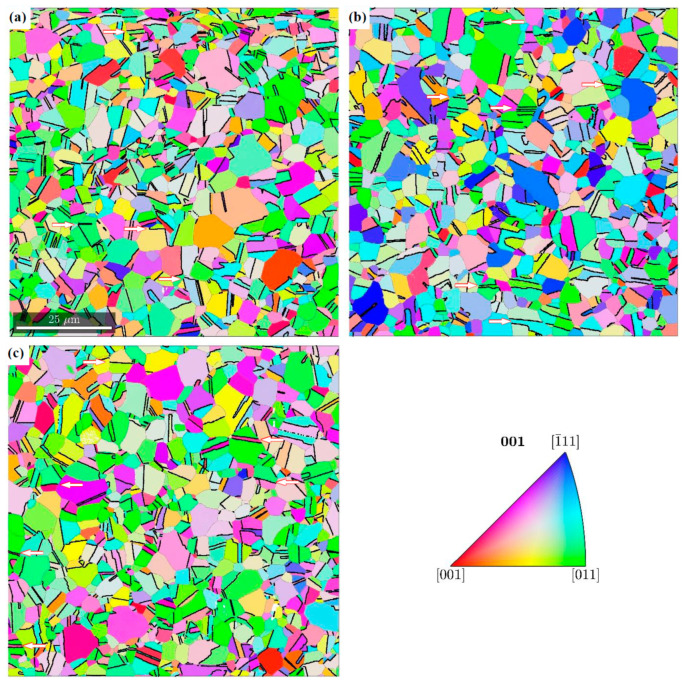
Orientation of austenite grains together with twin boundaries (thick black lines) in areas located at a distance of 4 mm from the fracture surface in specimens stretched at the temperature of 4.2 K (**a**), 77 K (**b**) and RT (**c**). The twins of special orientation are denoted by white arrows.

The misorientation analysis indicates that a significant part of the boundaries is formed in a twin relation. This misorientation occurs 50 times more often than the random one. The results of the synchrotron studies show that grains with the Copper orientation deform by twinning into the CopperT {5 5 2}<1 1 5> orientation and then rotate around the transverse direction (TD). As a result, the {1 1 1} planes locate at an angle of ±35° to the rolling direction (RD) (Figure 6). Many grains continue to rotate towards the Brass orientations which are stable for alloys with low intrinsic stacking fault energy. The results obtained are confirmed by EBSD investigations. In the representative areas, the twin boundaries are marked, in particular those, that were formed in the planes rotated around the TD direction and deflected by ±35° from the RD (Figure 7).

**Figure 8:**
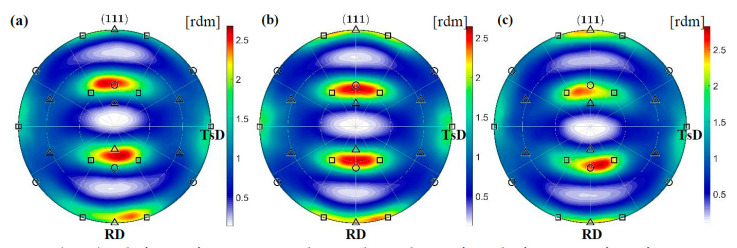
{1 1 1} pole figures for austenite in the area located 2 mm from the fracture surface of specimens stretched at the temperature of 4.2 K (**a**), 77 K (**b**) and RT (**c**). The Brass, Goss and Cu texture components are denoted by squares, circles and triangles, respectively.

The identified texture of the plastic matrix is partly preserved in the area of stress concentration, 2 mm from the fracture surface, where a decrease in the cross-section is visible. This time, however, deformation induced by tension dominates. As a result, the grains rotate more intensively around the ND || <0 1 1> direction, which allows the stable Brass orientation to be adopted (see Table 2). The PDF for {1 1 1} planes shows that the system does not preserve symmetry. The rotation to one of the Brass variants is preferred. The PDFs calculated from synchrotron measurements show that relatively few grains reach the final orientation (Figure 8) and a significant part is blocked during rotation. The existence of barriers which hinder slips will be examined using the EBSD method in the further part of the work.

### 3.4. Phase Transformation Controlled by Austenite

**Figure 9:**
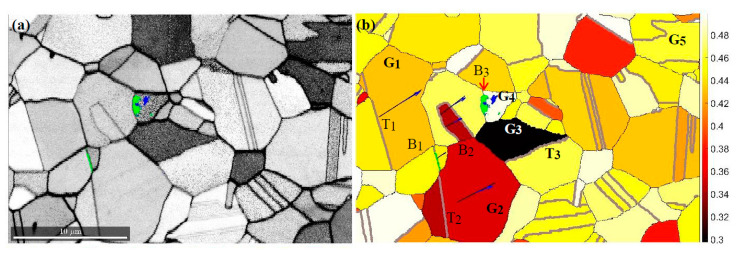

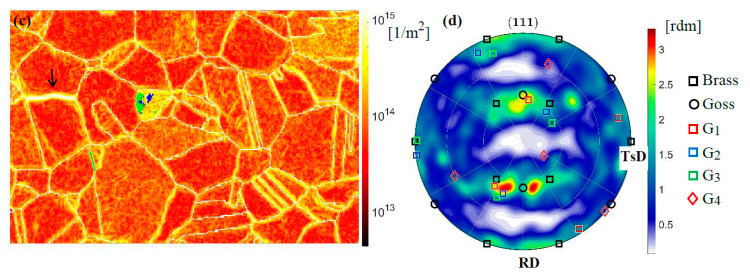
Fragment of the representative area at a distance of 4 mm from the fracture surface. (**a**) Image quality map (IQ) of austenite matrix with arising α’ martensite (blue) and ε martensite (green). (**b**) Map of maximal Schmid factors with corresponding traces of slip planes and slip directions, twin boundaries are denoted by thick grey lines. (**c**) Dislocation density distribution, the arrow indicates the band of strain localisation. (**d**) {1 1 1} pole figure for the entire representative area.

Away from the cross-section weakened by notches, the resulting stresses are too low to induce a significant plastic response of the material. Deformation occurs mainly through intensive twinning, and the limited dislocation movement does not stimulate phase transformation. As a result, only at the liquid helium temperature, along some boundaries and inside selected grains, are the hcp ε martensite as the precursor and the final α’martensite phase formed. The illustration constitutes one of the representative regions (see Figure 9a). Particularly susceptible to phase transformation are austenite crystallites oriented in relation to the tensile direction in a way that allows easy activation of one of the $\langle 1\;\bar{1}\;0\rangle \left\{1\;1\;1\right\}$ slip systems. These grains are identified by the Schmid factor close to 0.5. The coefficient has been calculated for 24 slip systems occurring in differently oriented crystallites of the studied region. The Schmid factor map for austenite with grain boundary overlay is illustrated in Figure 9b.

These results show that a group of grains arises, whose slip systems with the highest Schmid factor are formed along planes of similar orientation. The observed arrangement is due to the fact that the system tends to the stable Brass orientation (see Figure 9d). Grains at the initial Goss position reach it by rotation around ND || 〈1 1 0〉. As a result, slips occur in the planes {1 1 1} perpendicular to the sample surface, according to systems with the highest Schmid factors. In the final position, a twin boundary perpendicular to the tensile direction is often formed. The separated parts are two variants of the Brass orientation related to each other by a mirror plane. The above-mentioned path is covered by two grains G_1_ and G_2_ in the analysed area. As a result, along similarly oriented planes {1 1 1} with the highest resolved shear stresses, the boundaries B_1_ and B_2_ are formed. Inside the G_2_ grain, further slips occur, which generate clearly visible edge dislocations (Figure 9a). In addition, the twin boundaries $\langle 1\;1\;\bar{2}\rangle \left\{1\;1\;1\right\}$ T_1_ and T_2_ are formed almost perpendicularly to the tensile direction. Shearing along T_2_ continues to operate in the adjacent grain, resulting in the formation of ε martensite. Besides this, the resolved shear stresses from the G_2_ grain are further transferred to the G_3_ grain generating a twin boundary (T_3_). Consequently, the next grain assumes the stable Brass orientation. At the B_3_ boundary closing the area of coherency, slip planes generated in different systems with high Schmid factors intersect. This leads to the formation of ε martensite and α’ martensite. Details of the phase transformation are revealed by the dislocation density distribution analysis. The map obtained on the basis of the EBSD image shows significant concentrations of GNDs along the grain boundaries as well as inside the grain where intensive transformation takes place (G_4_). In the vicinity of martensite, edge dislocations in the plane (1 1 1) slightly deviated from the specimen surface are mainly formed. However, this process is not related to tension since the resolved shear stresses for this orientation are very small. It seems that the observed high dislocation density is introduced by the earlier rolling process, which then assisted by lowering temperature (4.2 K) may induce martensitic transformation. A similar effect occurs in other grains of the analysed area (e.g., G_5_). Thus, the orientations belonging to the γ fibre {1 1 1}||ND contribute to the initiation of martensitic transformation. Nucleation is activated by interaction with the deformation field intensively developing in the adjacent area. Synchrotron studies show that the main source of plastic strains is the system striving to the Brass orientation stable for 316L steel stretched transversely to rolling. Another clear effect of the registered tendency is a narrow, high-density dislocation zone formed in the G_1_ grain. It consists of two groups of edge dislocations. The first one is formed in the $\left(1\;\bar{1}\;1\right)$ plane with the Goss orientation, and the other one, of much higher density, in the $\left(\bar{1}\;1\;1\right)$ plane with the Brass orientation.

**Figure 10:**
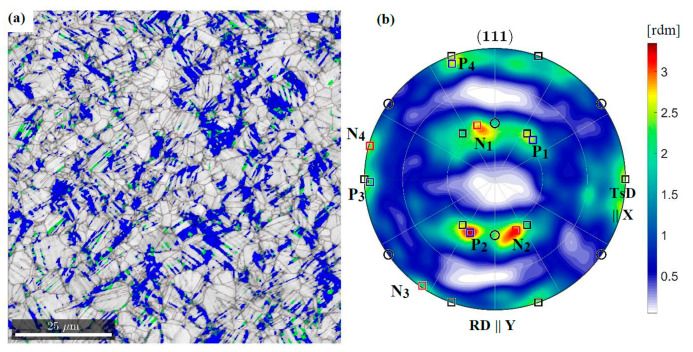
Microstructure of the representative region located 2 mm from the fracture surface. (**a**) Image quality map of austenite matrix with arising α’ martensite (blue) and ε martensite (green). (**b**) {1 1 1} pole figure for austenite and its two most preferred orientations (P, N).

**Figure 11:**
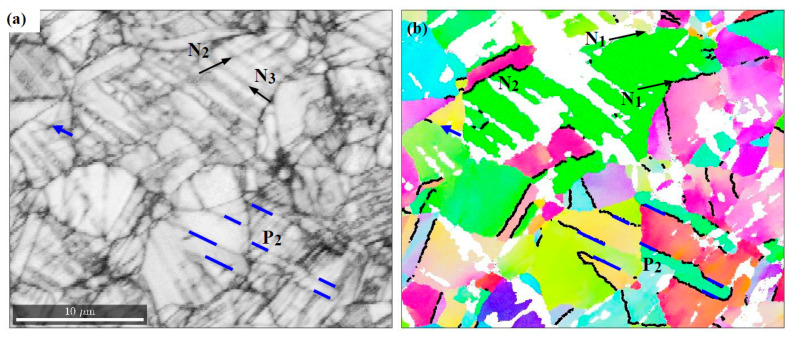

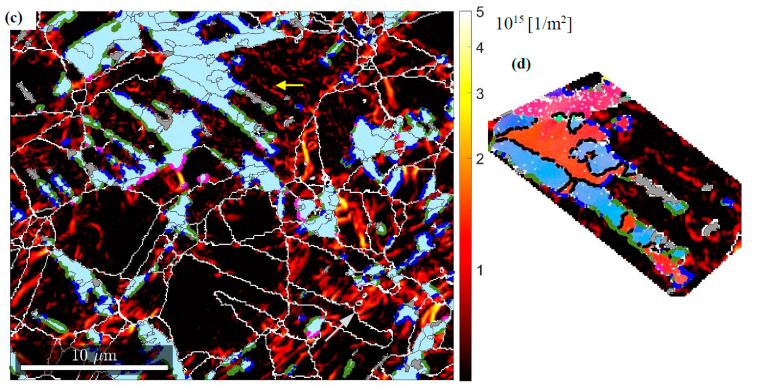
Details of the phase transformation. (**a**) IQ map (**b**) Orientation of austenite grains together with twin boundaries denoted by thick black lines. (**c**) GND map with disorientation at grain boundaries: Pitsch (blue), Kurdjumov-Sachs (green), Nishiyama-Wasserman (magenta), a band of screw dislocations is indicated by the yellow arrow, while dislocation pileups before a twin boundary by the grey arrow. In addition, the α’ martensite (light blue) and ε martensite (grey) are shown (**d**) Fragment in which α’ martensite orientations are depicted, twin boundaries are denoted by thick black lines.

In the area of stress concentration, 2 mm from the fracture surface, more intense shearing increases the content of α’ martensite and at the same time the transition phase of ε martensite. At liquid helium temperature, their volume fraction is 24.3% and 7.9%, and at liquid nitrogen temperature 17.9% and 7.2%, respectively. EBSD studies show that α’ martensite is formed in a non-random manner, fitting the austenitic matrix. In order to identify the phase transformation process, a representative area of the sample stretched at 4 K was examined (Figure 10a). The results show that austenite grains prefer two orientations (Figure 10b). They arise from the initial Goss position by positive (P) and negative (N) rotation around ND || <0 1 1>. The orientation P is close to the $\left\{0\;1\;1\right\}\langle \bar{2}\;1\;\bar{1}\rangle$ variant of the Brass orientation. However, it occurs less frequently than the other one. It turns out that the N grains do not reach the final position of the $\left\{0\;1\;1\right\}\langle 2\;1\;\bar{1}\rangle$ Brass variant, because intense slips cause the formation of martensite. This, in turn, prevents further rotation. The process is clearly visible in the case of the austenite grain separated by the {1 1 1} boundaries with the orientation N_1_, N_2_ (see the green grain in Figure 11b).

In order to describe the embedding of martensite inside the austenitic matrix, the GND density distribution was determined. Separation of the aforementioned grain along the boundaries N_1_ ${\left(\bar{1}\;1\;1\right)}_{\gamma }$ and N_2_ ${\left(1\;1\;1\right)}_{\gamma }$ results in the presence of high density edge dislocations in these planes. Besides this, the image quality map (IQ) shows clearly visible traces along the N_3_ ${\left(1\;1\;\bar{1}\right)}_{\gamma }$ planes. They identify the α’ and ε martensite bands, as well as such areas where the phase transformation occurs only locally. Isolated grains of α’ martensite are formed at the intersection of the N_2_ and N_3_ plains. This suggests a classic way of transformation [33], according to which α’ martensite is a product of intersecting bands of ε martensite. Then, the austenite/α’ martensite boundaries are built using the screw dislocations $\frac{1}{2}{\left[0\;1\;\bar{1}\right]}_{\gamma }$. The performed GND analysis shows their high density in the areas of phase transformation. They pile up in the plane ${\left(\bar{5}\;2\;2\right)}_{\gamma }$, which results in formation of the boundary with α’ martensite. The slip along ${\left[0\;1\;\bar{1}\right]}_{\gamma }$ continues inside the new phase leaving behind the screw dislocations $\frac{1}{2}{\langle 1\;\bar{1}\;1\rangle }_{\alpha }$ piled up in the plane ${\left\{2\;1\;\bar{1}\right\}}_{\alpha }$. The formation of boundaries in terms of ${\left\{\bar{5}\;2\;2\right\}}_{\gamma }||{\left\{2\;1\;\bar{1}\right\}}_{\alpha }$ indicates that the α’ martensite assumes the Pitsch disorientation defined by the following relations ${\left(1\;0\;1\right)}_{\gamma }∥\;{\left(1\;1\;1\right)}_{\alpha }$ and ${\left[0\;1\;0\right]}_{\gamma }∥\;{\left[\bar{1}\;1\;0\right]}_{\alpha }$. The EBSD results show that along the austenite/α’ martensite boundaries, the Pitsch relationship smoothly transforms into the next one, i.e., Kurdjumov-Sachs’. Then, around the direction determined by the developing screw dislocations, there is a small rotation, which leads to the following orientation dependence ${\left(1\;0\;1\right)}_{\gamma }∥\;{\left(1\;1\;1\right)}_{\alpha }$ and ${\left[1\;1\;\bar{1}\right]}_{\gamma }∥\;{\left[0\;1\;\bar{1}\right]}_{\alpha }$. In this way, better fitting between both lattices is obtained. Locally, in areas of strain concentration, α’ martensite is formed, which is oriented relative to the austenitic matrix according to the Nishiyama-Wasserman relation ${\left(1\;0\;1\right)}_{\gamma }∥\;{\left(1\;1\;1\right)}_{\alpha }$ and ${\left[1\;1\;\bar{1}\right]}_{\gamma }∥\;{\left[0\;1\;\bar{1}\right]}_{\alpha }$.

**Figure 12:**
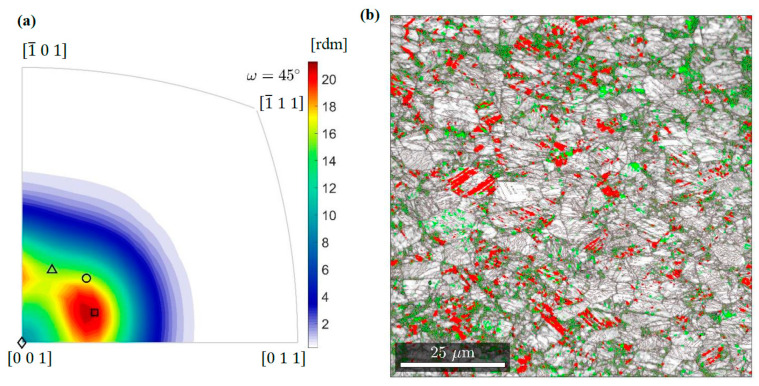
(**a**) MDF for austenite/α’martensite boundaries in the area 2 mm from the fracture surface with the key relations: Pitsch (square), Kurdjumov-Sachs (circle), Nishiyama-Wasserman (triangle), Bain (diamond). (**b**) Microstructure of the specimen area at the fracture surface: image quality map of α’ martensite with grains of austenite (red) and ε martensite (green).

The misorientation distribution function (MDF) reveals frequencies with which the individual relationships are assumed (Figure 12a). This is presented in the axis/angle convention, according to which the martensitic reference frame arises from the austenitic one by rotation around an axis by a certain angle. For example, the well-known Bain disorientation is obtained by a rotation around an austenite cell edge by 45 degrees. Thus, in the stereographic projection, it is represented by a point in the origin of the coordinate system. Considering the cross-section of the fundamental area of disorientation space for 45 degrees, in addition to the Bain relation, other relations most often formed in 316 L steel can be depicted. Rotation angles of disorientations with high frequencies do not differ much from 45°—the Pitsch, Kurdjumov-Sachs and Nishiyama-Wasserman orientation relations are characterized by 45.99°, 42.85°, and 45.99°, respectively. Hence, the combination of all relationships in one section of space allows the preferences identified at the temperature of liquid helium to be clearly and correctly shown. It turns out that the Pitsch disorientation becomes dominant. A similar preference is observed at 77 K while at room temperature the Kurdjumov-Sachs or Nishiyama-Wasserman relations are most often formed.

Besides the generation of special boundaries, the new phase is fitted by twinning. In the studied representative area (Figure 10), 40% of boundaries arise in the twin relation $\left\{1\;1\;2\right\}\langle \bar{1}\;\bar{1}\;1\rangle$. EBSD investigations show that martensite is also formed in grains with stable orientation determined by the P Brass variant. However, the mechanism for generating a new phase is different. It is associated with breaking the boundary of the stable austenite grain through piled up dislocations (Figure 11c). The barriers along which accumulations occur may run through several grains due to the tendency to form coherent regions. This behaviour is similar to that observed earlier, in the zone of lower stress 4 mm from the fracture surface (comp. Figure 9b).

In the vicinity of the fracture surface, α’ martensite becomes the dominant phase (Figure 12b). Strong deformations mean that only 17% of the boundaries are twins. Thus, the α’ martensite/α martensite boundaries are largely random. In addition, particles of ε martensite or austenite often accumulate along them. Boundaries formed in this way facilitate crack propagation.

## 4. FEM Analysis of Fracture in Two-Phase Continuum

### 4.1. XFEM Method

When modelling fracture by means of XFEM, discontinuous functions are added to the standard polynomial functions to accommodate crack opening and penetration of the crack into the element located in front of the crack tip. The classical finite element method (FEM) was expanded by Belytschko and Black [34] to the extended version, called XFEM. The presence of discontinuities is essentially covered by the introduction of enriched functions, as well as supplementary degrees of freedom. The initiation of the macro-crack is, in light of the XFEM method, related to the onset of deterioration of the cohesive response of the enhanced element. The process of deterioration starts when the representation of the stresses or the strains meets the criterion of crack initiation. The following crack initiation criteria, based on the Abaqus/Standard integral models, are available [35]: the maximum principal stress or the maximum principal strain criterion, the maximum nominal stress or the maximum nominal strain criterion, the quadratic traction-interaction criterion, and the quadratic separation-interaction criterion. In order to simulate fracture at extremely low temperatures, the maximum principal strain criterion has been used:

(1) $$f=\left\{\frac{\langle \varepsilon_{\max}\rangle }{\varepsilon_{\max}^{0}}\right\}$$

Here, *f* is the criticality ratio, $\varepsilon_{\max}^{0}$ stands for the maximum allowable principal strain, and the Macaulay bracket signifies that the compressive strain does not contribute to fracture. The initiation of fracture takes place when the above defined ratio (Equation (1)) is equal to 1.

### 4.2. Kinetics of Phase Transformation

**Figure 13:**
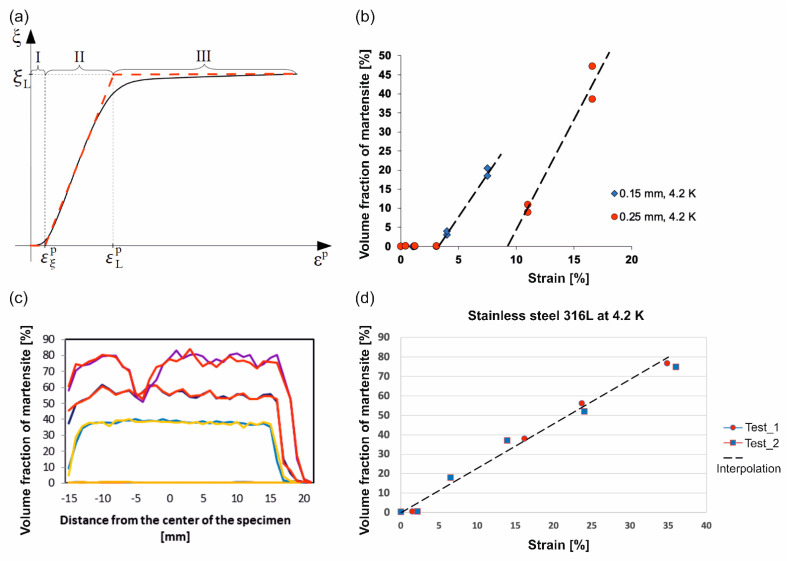
Volume fraction of martensite as a function of plastic strain: (**a**) schematic dependence, (**b**) magnetometer measurement for grade 316L stainless steel (Table 3) samples with different thickness, (**c**) martensite distribution along the sample axis for 316L steel defined in Table 1, as a basis for the relationship presented in graph (**d**).

The plastic strain induced *γ-α’* phase transformation in metastable materials (stainless steels) consists in rapid transition from the parent face- centred cubic phase to the secondary body-centred cubic phase. It is usually distinguished from the stress assisted phase transformation, characteristic of the shape memory alloys. The plastic strain induced fcc-bcc phase transformation occurs in a wide range of temperatures, typically below the threshold temperature *M_d_* [36,37]. The phase transformation in stainless steels can be easily activated both in liquid nitrogen (at 77 K), and in liquid helium (at 4.2 K). In the latter case, it is usually accompanied by the so-called discontinuous plastic flow (serrated yielding). The phase transformation kinetics is reflected by the relation between the rate of the volume fraction of the secondary phase and the plastic strain rate. Originally, kinetics of the low temperature plastic strain induced *γ-α’* phase transformation was developed by Olson and Cohen [10]. It is illustrated by a typical sigmoidal curve (Figure 13) that indicates the evolution of the volume fraction of the secondary phase (*ξ*) as a function of plastic strain (*ε^p^*). The Olson and Cohen approach was based on a number of assumptions, including the shear band intersection mechanism. The volume fraction of shear bands was determined by the following equation:

(2) $$\varsigma =1-e^{-\alpha \varepsilon^{p}}$$

where *α* denotes a parameter representing the rate of shear band formation. Furthermore, a constant average volume of the shear band *υ* was assumed. Thus, the number of shear bands *N*, and the number of shear band intersections *N_χ_*, read:

(3) $$N=\frac{\varsigma }{\upsilon }\;;\;N_{\chi }=kN^{n}$$

The shear bands were approximated by thin plates of average diameter *d*, which allows the parameter *k* to be computed effectively. Evolution of the number of martensitic embryos *N_α_* has been associated with the probability that a shear band intersection will generate the embryo *p_α_,* which leads to the following equation:

(4) $$dN_{\alpha }=p_{\alpha }N_{\chi }$$

where the probability has been determined as a Gaussian function of temperature *T*. Another assumption was related to the morphology and constant volume of the martensitic site *υ_α_*. Consequently, the evolution of the volume fraction of *α’* martensite reads:

(5) $$d\xi =(1-\xi )\upsilon_{\alpha }dN_{\alpha }$$

By combining Equations (2)–(5) and performing suitable integration, one obtains:

(6) $$\xi =1-e^{-\beta {\left(1-e^{-\alpha \varepsilon^{p}}\right)}^{n}}\;;\;\beta =\frac{\upsilon_{\alpha }kp_{\alpha }}{\upsilon^{n}}$$

Eventually, the Olson-Cohen kinetics contains essentially three parameters: two of them are temperature dependent (*α*, *β*) and *n* is a fixed exponent.

In view of the fact that at extremely low temperatures the phase transformation process is much steeper in terms of *dξ/dε^p^* than at room temperature, three stages can be distinguished: low transformation rate below the threshold *ε_ξ_* (I), high transformation rate with a nearly constant transformation slope *dξ/dε^p^* (II), and asymptotically vanishing transformation with the rate decreasing to 0 (III) (see Figure 13a). The volume fraction of martensite usually reaches a maximum of *ξ_L_* during Stage III. The phase transformation at extremely low temperatures (4.2 K or 77 K) manifests itself by the fact that the vertical part (II) of the sigmoidal curve remains within small or moderately large strains. Consequently, the kinetics can be formulated in the framework of small strains (the Cauchy strain measure).

In view of the particularly fast transformation process at extremely low temperatures (within Stage II), linear kinetics has been developed [38] in the following form:

(7) $$\dot{\xi }=A\left(T,\sigma \right){\dot{\varepsilon }}^{p}H\left(\varepsilon^{p}-\varepsilon_{\xi }^{p},\xi_{L}-\xi \right)$$

where dot denotes the time derivative, or in a more general, multiaxial form:

(8) $$\dot{\xi }=A\left(T,\sigma_{ij}\right)\dot{p}H\left(p-p_{\xi },\xi_{L}-\xi \right)$$

Here, *σ_ij_* denotes the second order covariant stress tensor, *p* means the accumulated plastic strain, *A* is a function of temperature and stress state, whereas, *p_ξ_* denotes the threshold strain (to trigger the phase transformation). Furthermore, *ξ_L_* stands for the maximum martensite content, and *H* represents the Heaviside function. For a complex, multiaxial stress state, it is assumed that the fcc-bcc phase transformation is driven by the accumulated plastic strain *p* (the Odqvist parameter):

(9) $$p=\int_{0}^{\tilde{t}}\sqrt{\frac{2}{3}d{\dot{\varepsilon }}_{ij}^{p}:d{\dot{\varepsilon }}_{ij}^{p}}dt$$

For isothermal processes and small variation of stress, the phase transformation kinetics reduces to:

(10) $$d\xi =Adp\;;\;p\geq p_{\xi }\;,\;\xi_{L}\geq \xi$$

where *A* reflects the volume fraction of martensite as a function of plastic strain during Stage II. The threshold strain *p_ξ_* and the constant *A* depend on temperature and the chemical composition of the steel [39]. At extremely low temperatures, the initial, temperature driven, spontaneous phase transformation, triggered by the existing potential nucleation sites, is usually taken into account. It occurs below the temperature *M_s_*. As the temperature *M_s_*, calculated for the stainless steel under consideration by means of the formulae developed by G.H. Eichelman and F.C. Monkman, amounts to 13.3 K or 82.6 K, respectively, it is possible that at the temperature of liquid helium some limited spontaneous phase transformation might occur. Thus, the final volume fraction of the secondary phase consists of two components, the contribution induced by spontaneous phase transformation $\xi_{0}$ and the constituent due to plastic strains Δξ:

(11) $$\xi =\xi_{0}+\Delta \xi \;;\;\Delta \xi =A\Delta p$$

The measurements of the amount of the secondary phase versus strain for grade 316L at 4.2 K are shown in Figure 13. For the reference material the amount of martensite has been measured by magnetic methods [40]. The set-up (Figure 13b), used to measure the magnetization (M), was composed of a superconducting coil and contained temperature stabilization. Suitable instrumentation to measure the magnetization, as well as the magnetic field (H), was included. The measuring technique was based on the assessment of the magnetization by means of the magnetic flux induced by the sample inside the measuring coil. To measure the magnetic flux, both the VSM (vibrating sample magnetometer) method and the extraction magnetometer (EM) method were applied. In order to calculate the flux, the reciprocity theorem has been used.

For the material under consideration in the present paper (see Table 1), the volume fraction of martensite has been quantified by means of a ferritscope. Figure 13c shows the distribution of the secondary phase along the sample axis at four stages of the deformation process (the upper profiles correspond to fracture). The measurements were carried out with a ferritscope at 37 points along the axis of the sample. It is worth pointing out that the measurements were conducted on the upper and on the lower surface of the sample in order to perform a cross-check of the data.

Generally, given the assumed linearity of the second stage of the transformation curve (II), the increment of the volume fraction of the new phase is directly proportional to the increment of the plastic strain in the isothermal conditions (*T = const*). The threshold and the slope of the phase transformation kinetics were identified for 316L stainless steel with the chemical composition determined in Table 1: *p_ξ_* = 0; *A* = 2.29.

### 4.3. Constitutive Model

**Figure 14:**
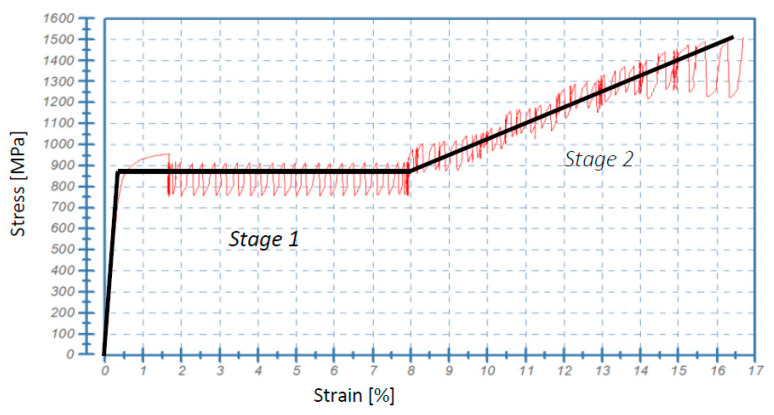
Identification of parameters of the constitutive model for grade 316L stainless steel (cf. Table 1) at 4.2 K (Stage I: no hardening, Stage II: linear hardening).

As the material parameters required in the constitutive model are determined by means of suitable experiments, at first, the stress-strain relationship for the material under consideration was obtained (Figure 14), and then the relevant constitutive representation was selected. The stress-strain oscillations were neglected in the simulations of fracture, and the adopted material model used in the numerical simulations was elastic-plastic with linear isotropic hardening. The model was based on the assumption that each “serration” (oscillation of stress as a function of strain or time) contains elastic loading followed by smooth plastic flow, represented by the flow stress that can be identified in the upper part of serration. As soon as the flow stress is reached, smooth plastic flow takes place until the abrupt drop of stress occurs. In the first part of the stress-strain curve (Stage I, Figure 14), the initial flow stress within each serration is identical, which leads to the conclusion that—as an approximation—a perfectly plastic model can be used:

(12) $$f(\sigma_{ij})=\sigma_{i}-\sigma_{0}(T)\;;\;\sigma_{i}=\sqrt{\frac{3}{2}s_{ij}s_{ij}}$$

(13) $$d\varepsilon_{ij}^{p}=d\lambda \frac{\partial f(\sigma_{ij})}{\partial \sigma_{ij}}$$

where the yield function $f(\sigma_{ij})$ stands for the dissipation potential for plasticity, $s_{ij}$ denotes the deviatoric stress, and $\sigma_{0}$ is the flow stress (the same for each serration), that depends on the temperature *T*. However, in the second part of the stress-strain curve (Stage II), where the hardening threshold has been reached, nearly perfect linear hardening is observed. Here again, the initial values of the flow stress within each serration form a linear approximation of hardening. Within Stage II, the elastic-plastic model with linear isotropic hardening has been used:

(14) $$f(\sigma_{ij})=\sigma_{i}-\sigma_{R}(T)\;;\;\sigma_{R}=\sigma_{0}+R$$

where *R* stands for the isotropic hardening parameter, obeying the following evolution law:

(15) $$dR=C_{R}dp$$

where *C_R_* denotes the hardening modulus. Thus, it has been assumed that the effect of the stress-strain oscillations on the macro-crack propagation is minor, and the phase transformation (evolution of the microstructure) is crucial. Finally, the following parameters of the material model were identified:

### 4.4. Numerical Model

**Figure 15:**
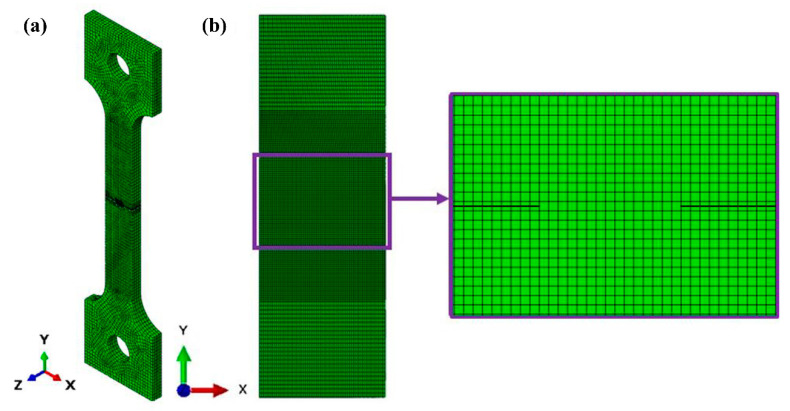
Finite element model of the sample containing two symmetric cracks on either side: the initial (**a**) and final geometry (**b**).

The numerical model has been built using the XFEM ABAQUS software (Abaqus/CAE 2017, Dassault Systemes Simulia Corp., Johnston, Rhode Island, USA), and supplemented by suitable boundary conditions and external loads, in the form of forced displacements causing an extension of the sample. The objective of each numerical test was to simulate macro-crack propagation under variable loads. Discretization of the model was carried out by means of the automatic mesh generator, at the heads of the sample, and the mapped meshing technique in the macro-crack region. The mesh initially contained some 11,112 elements (C3D8R) and 14,780 nodes (Figure 15a). As the number of elements was relatively small, after the initial simulations, the studied area was confined, and the mesh density was increased. Finally, some 164,400 elements were applied (Figure 15b).

Elimination of the upper and lower part of the sample enabled more accurate calculations to be performed, but did not affect the solution in the surroundings of the macro-crack. This is mainly due to the strong localization of the inelastic deformations. The eight-node elements with three degrees of freedom at each node and reduced integration (C3D8R) are characterized by a high degree of accuracy. This is achieved by removing false modes of deformation due to the application of higher order polynomial representation [41]. The numerical model with the implemented mesh for the entire structure is shown in Figure 15.

The main objective of the numerical analysis is to simulate the crack propagation across the sample and to analyse the stress and the strain fields, as well as the phase transformation evolution, in the vicinity of the crack tip. The numerical results were cross-checked with the evolution of microstructure observed by means of the EBSD technique and X-ray diffraction using synchrotron radiation.

### 4.5. Numerical Results

**Figure 16:**
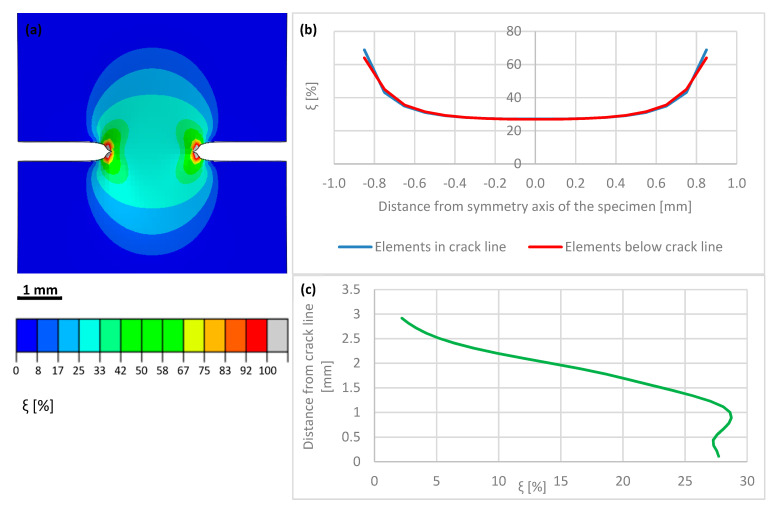
Volume fraction of martensite at the beginning of double crack propagation (Stage I): (**a**) distribution on the sample surface (**b**) values along the crack path (**c**) values along the sample axis.

**Figure 17:**
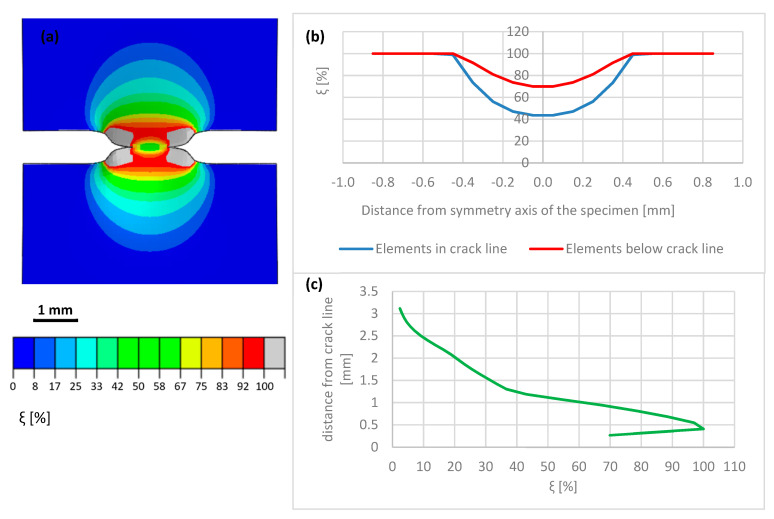
Intermediate stage (II) of double crack propagation: (**a**) map of volume fraction of martensite (**b**) tip-to-tip distribution of the secondary phase (**c**) ξ-profile along the axis of the sample (perpendicular to the tip-to-tip axis).

**Figure 18:**
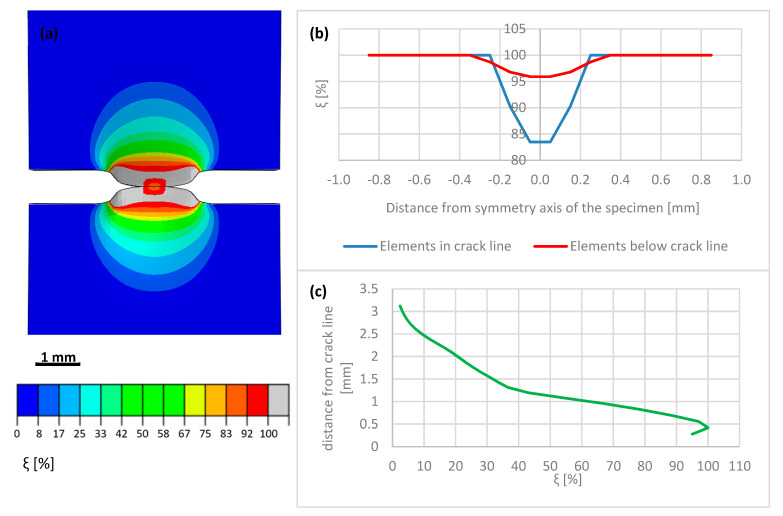
Final stage (III) of double crack propagation: (**a**) map of volume fraction of martensite (**b**) tip-to-tip distribution of the secondary phase (**c**) ξ-profile along the axis of the sample (perpendicular to the tip-to-tip axis).

The macro-crack evolution has been subdivided into three stages: the initial stage (I) of macro-crack onset, the intermediate stage (II) of advanced macro-crack, and the final stage (III) of macro-crack propagation through thickness. During Stage I, strong localization of the plastic strains in the vicinity of the crack tip is observed. The increment of the volume fraction of the secondary phase is strictly related to the increment of the accumulated plastic strain by means of transformation kinetics. A map of the volume fraction of the secondary phase during the initial stage of macro-crack propagation is shown in Figure 16a. In addition, the distribution of the volume fraction of the secondary phase along the horizontal axis linking both crack tips (anticipated macro-crack trajectory) is illustrated in Figure 16b, whereas the martensite fraction in the direction perpendicular to the macro-crack trajectory (i.e., parallel to the axis of the sample) is shown in Figure 16c. It is worth pointing out that—already in the initial stage of the process—the volume fraction of martensite reaches some 65–70% in the vicinity of the crack tip. Between the macro-crack tips, the martensite content drops to a level well below 30%, reaching in the middle of the sample some 27%. Additionally, the volume fraction of the secondary phase, traced in the direction parallel to the sample axis, follows a steep profile and reaches around 2.5% at a distance of 3 mm from the tip-to-tip axis. During Stage II, corresponding to intermediate crack length, the phase transformation process is already well advanced (Figure 17a). The volume fraction of the secondary phase reaches 100% in the vicinity of the crack tips. In the bridge between the cracks (tip-to-tip axis), the volume fraction of martensite reaches 40% (Figure 17b), which indicates well advanced phase transformation in the middle of the sample. When searching in the direction normal to the tip-to-tip axis (Figure 17c), the volume fraction of the secondary phase drops from 100% to 60% at 1 mm distance and, eventually, to 3% at a distance of 3 mm. Finally, during Stage III, the macro-cracks fully penetrate the sample and meet in the middle (Figure 18a). Nearly the whole zone on either side of the macro-crack is transformed to 100%. Only in the middle of the sample, the amount of secondary phase drops to around 85% (Figure 18b).

The finite elements penetrated and split by the macro-crack are unloaded due to the crack opening (the stored energy is released in the process of creation of free surfaces), which is reflected by lower intensity of the phase transformation (blue line in Figure 18b). In particular, the martensite fraction in the middle of the sample reaches around 83%, whereas, in the elements located just below the macro-crack trajectory, the volume fraction of the secondary phase reaches more than 95%. The plot of martensite fraction in the direction normal to the tip-to-tip axis indicates 100% in direct proximity of the macro-crack, then a steep decrease to 60% at a distance of 1 mm, and a final drop to 3% at 3 mm distance. The results clearly show intensive phase transformation in the zone located in the proximity of the macro-crack trajectory, and fast decay of the volume fraction of the secondary phase when leaving the crack zone. At a distance of more than 3 mm, the material essentially does not undergo phase transformation.

It is worth pointing out that the applied elastic-plastic constitutive model, based on linear hardening and accompanied by the phase transformation kinetics converting accumulated plastic strain to volume fraction of the secondary phase, offers a quite realistic insight in the physical phenomena that take place into the material microstructure. In order to improve the constitutive model, a fully coupled plasticity-phase transformation non-linear version might be used. However, the results from the qualitative point of view will be the same.

## 5. Comparison of Experimental and Numerical Results

### 5.1. Distribution of the Secondary Phase in the Macro-Crack Proximity

**Figure 19:**
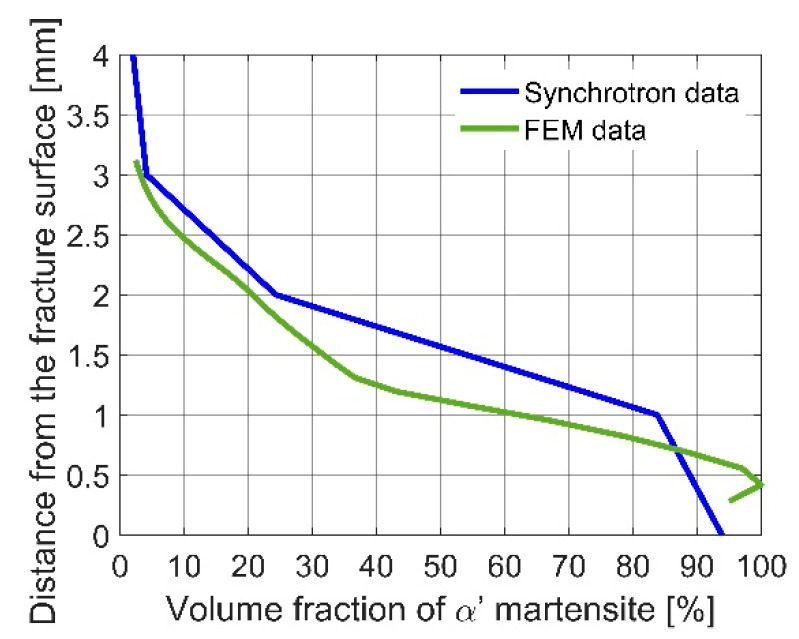
Synchrotron measurements compared with FEM results.

The numerical results were obtained by means of the FE model, based on the XFEM approach. The model has been calibrated by using the stress-strain diagrams (Figure 14) obtained from the experiments performed in liquid helium (4.2 K). It is worth pointing out that the stress-strain curve, recalculated from the force-elongation diagram contains the hardening modulus associated with the plastic strain induced fcc-bcc phase transformation. In the numerical model, linear hardening has been assumed for simplicity, which does not exclude more sophisticated non-linear hardening models from being implemented in future work. The main feature of the model consists in its ability to trace the trajectory of the macro-crack in a way that is, to some extent, mesh independent. The maps containing the distribution of martensite content are shown in Figure 16, Figure 17 and Figure 18. They were obtained on the basis of accumulated plastic strain according to Equation (11). The highest values of the volume fraction of martensite are observed in the vicinity of each crack (on either side of the sample). Moreover, both cracks are connected by the annular zone of the enhanced accumulated plastic strain, containing a much less loaded zone in the middle of the sample. This is confirmed by the profiles of the volume fraction of martensite traced along the path linking both cracks, as well as in the direction perpendicular to the macro-crack trajectory (parallel to the axis of the sample, Figure 17). Direct verification of the numerical results consists in comparing the distribution of the martensite volume fraction along the symmetry axis of the broken sample (Figure 18c) with the curve obtained from the synchrotron studies (Figure 4a). It turns out that the theoretical function is similar to the experimental one. In particular, the volume fraction of martensite achieves a very high value, around 100%, in the proximity of the crack tip. This content is considerably reduced with the distance from the crack tip (Figure 18c), to reach practically 0% at a distance of 4 mm. Simultaneously, the austenite volume fraction shows inverse behaviour, which means the increase in its content from practically 0% close to the crack tip, to around 100% at a distance of 4 mm. The cross-check of the numerically obtained distribution of the martensite volume fraction with the microscopic observations shows fairly good convergence (see Figure 19). The differences that occur in the middle field (1–2 mm) are attributed to the constitutive model, which includes the kinetics of the phase transformation, however, full coupling between the phase transformation and the plasticity, including the evolution of the tangent stiffness in the course of the process, has not been taken into account. Thus, the hardening model is not fully coupled to the strain induced phase transformation, which may affect the results of the numerical analysis especially in the steep part of the martensite fraction versus distance curve. Improvement of the constitutive model to a fully coupled version is anticipated in the near future.

### 5.2. Microstructure Evolution during Fracture

**Figure 20:**
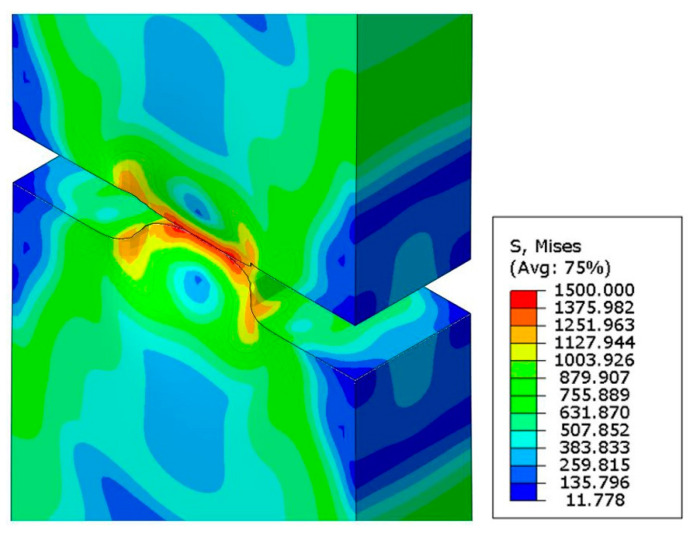
Von Mises stresses in the final stage of fracture.

Symmetrical notches introduce the variable stress distribution along the specimen axis. As a result, at the temperature of liquid helium, the yielding stage is virtually eliminated (see Figure 3). Strong plastic deformations are combined with the formation of the new phase responsible for strengthening the material. Similar conditions occur in the front of the initiating macro-crack, where the volume fraction of martensite reaches 27%. Further stages of the fracture process proceed in a strongly three-axial deformation state. As a result, the crack develops in a medium dominated by the hard phase, and 100% martensitic transformation occurs at the very tip. The distribution of the von Mises stress together with the spatial deformation arising at the moment of fracture is shown in Figure 20.

The discussed conditions in the individual stages of macro-crack development can be correlated with those that occur in subsequent areas along the axis of the broken specimen. At a distance of 2 mm from the fracture surface, the degree of phase transformation is similar to that identified during crack initiation. Thus, the microstructural analysis performed earlier (Section 3.4, Figure 10) reveals, that in the initial stage of macro-crack development, band-shaped martensite coherently bonded to the austenitic matrix is formed. The new phase is fitted through special disorientations as well as twinning. Thus, hard α’ martensite particles act as the reinforcing phase in the ductile austenitic matrix. The microstructure registered at the fracture surface (Figure 12b) shows the conditions in which the macro-crack propagates after covering half the path. A significant reduction of twins means that, when combining martensitic grains generated at different stages of loading, random boundaries arise. The ε martensite often accumulates along these boundaries. This creates channels for easy crack propagation. Hence, the growing macro-crack is accompanied by a network of micro-cracks. They are partly trapped by austenite remains and larger clusters of ε martensite.

## 6. Discussion

The microstructure analysis in the vicinity of the crack tip and along the crack path is of primary importance from the point of view of understanding the mechanisms of macro-crack propagation. This problem becomes even more crucial at extremely low temperatures because of specific circumstances that accompany the macro-crack onset and propagation.

Stainless steels belong to the materials commonly used at extremely low temperatures because of their excellent mechanical and physical properties, including ductility. The present paper deals with one of the most popular stainless steels, grade 316L. The chemical composition contains enhanced chromium (usually 16–18%) and nickel (usually 10–14%) content. Such a composition does not eliminate the risk of phase transformation, therefore, the stainless steels belonging to the 316L family undergo the plastic strain induced fcc-bcc phase transformation at extremely low temperatures. This implies possible phase transformation in the proximity of the macro-crack tip, and quite important implications for further macro-crack propagation. Assuming the Dugdale model of a macro-crack in the elastic-plastic continuum, a butterfly plastic zone occurs in front of the crack tip and the fcc-bcc phase transformation is expected. The EBSD investigations, as well as fine measurements performed by using the synchrotron radiation, show that the microstructure in the vicinity of the crack tip can be almost entirely transformed from austenite to martensite (Figure 4a and Figure 12b). This implies the conditions of further macro-crack propagation to be rather fragile than ductile, due to the fact that the martensitic phase at extremely low temperatures behaves in a nearly elastic way (the yield stress is quite high). Analysis of the conditions of macro-crack propagation in stainless steels at extremely low temperatures shows, that the plastic zone in front of the crack tip becomes nearly entirely transformed (70% of martensite), which implies the existence of two-phase continuum with domination of the secondary phase. Moreover, from the point of view of properties of two-phase continuum represented by tangent stiffness computed by means of the mean field methods (homogenization), there is an interchange of roles: the former matrix (austenite) has to be treated like inclusions, and the former inclusions (martensite) constitute the matrix. This obviously leads to rather strong complications in terms of mathematical description of the properties of two-phase continuum in front of the crack tip.

As the symmetric sample contains two cracks (facing each other on both sides of the sample), it is interesting to analyse the distribution of the secondary phase between the crack tips, which is shown in Section 4.5 (Figure 16, Figure 17 and Figure 18). In particular, it is clear that initially the volume fraction of the new phase decreases in the space between the crack tips, however, when the crack tips propagate and get closer to each other, the distribution of the secondary phase changes and the level of volume fraction of martensite becomes more uniform. This means that the fracture should have brittle features, and this effect has been confirmed by suitable microscopic analysis. Another interesting phenomenon has been observed in the direction perpendicular to the trajectory of both cracks (parallel to the axis of the sample). Apparently, the volume fraction of the secondary phase reaches the maximum at a certain distance (typically 0.5 mm) from the trajectory of both cracks (annular plastic zone between the crack tips) and significantly decreases, which leads to a low value of 20% at a distance of some 2 mm. The obtained dependence has been confirmed by the analysis of the microstructure (see Figure 10). This indicates another interesting feature of the zone surrounding both crack tips. Namely, at a distance of 2 mm, the microstructure is weakly transformed, and the primary phase dominates with only a small amount of the secondary phase. Thus, the classical microstructure containing the austenitic matrix and rare martensitic inclusions is observed. In conclusion, the macro-crack induces strong phase transformation in the direct vicinity of the crack tip, however, at a sufficient distance from the macro-crack trajectory a nearly untransformed microstructure is observed.

One interesting point is whether the phase transformation in front of the crack tip promotes the macro-crack propagation or not. Given the fact that the austenite and martensite are coherently bonded due to non-random disorientations, any presence of a secondary elastic phase should essentially reduce the possibility of macro-crack propagation (strong obstacle in front of the crack tip). However, when the phase transformation has a massive character (more than 70% of microstructure transformed), the probability of brittle fracture increases, which leads to favourable conditions of fast macro-crack propagation. Thus, a fast and massive phase transformation process in front of the crack tip leads to a much higher probability of fracture, which has to be taken into account when designing the structures operating in cryogenic conditions. Moreover, a strong indication to select materials with a limited phase transformation should be expressed, since a limited phase transformation in front of the crack tip (less than some 30% of the new phase) might enhance the fracture toughness.

## 7. Conclusions

In the present paper, the conditions of fracture in metastable stainless steels were discussed. In particular, one of the most popular stainless steels, AISI 316L, commonly applied at extremely low temperatures, was tested against fracture. The symmetric samples containing two artificially initiated cracks (facing each other on both sides of the sample) were used in order to advance the fracture at the temperature of liquid helium (4.2 K) or liquid nitrogen (77 K). As the stainless steel is metastable, the plastic strain induced phase transformation from fcc austenite to bcc martensite accompanies the fracture. The volume fraction of the secondary phase varies both between the cracks, and in the direction perpendicular with respect to crack trajectory. The evolution of microstructure was investigated by means of EBSD and globally by synchrotron X-ray diffraction. In particular, the orientation distribution function for the austenitic phase at various distances from the fracture surface was studied. The type of microstructure, including the type of boundaries between the grains as well as the distribution of both phases (primary and secondary), the misorientation distribution function for the austenite/martensite boundaries and the microstructure at the fracture surface, were thoroughly investigated for the samples tested at the liquid helium temperature. The results were discussed in the context of analogical examinations performed at higher temperature levels (77 K and 293 K). Three mechanisms of strain-induced phase transformation were revealed. Each of them is associated with austenite striving for the Brass orientation which is stable for metals and alloys with low stacking fault energy when subjected to tension in the direction transverse to rolling. The first mechanism is activated in the zone of low stress, not exceeding the yield point. Thus, it requires the presence of strong initial deformations induced by rolling. The martensitic transformation starts at the boundary, along which the strained austenite grain meets the coherent region with intense slips associated with rotation to the Brass orientation. Further mechanisms are activated in the zone of triaxial deformation induced by symmetrical notches. The increase in stress causes more intensive rotation of the austenite grains. Meanwhile, at the temperature of liquid helium, their accommodation by means of dislocation gliding is difficult. This leads to inhomogeneous twin shears, i.e., the formation of ε martensite bands, whose crossing generates the final, hard phase—α’ martensite. In this way, rotation of the austenite grains is blocked and only a small part of them reaches the stable Brass orientation. The mechanism is uncovered in the EBSD studies. The confirmation constitutes the untypical texture revealed in synchrotron investigations. Besides this, it turns out that some austenite grains achieve the final Brass orientation by twinning. Then, they act as barriers piling up dislocations. Breaking them leads to discontinuous flow during which martensite grains are generated. So, under the same conditions, two different mechanisms for creating martensite arise. Regardless of which one is activated, the new phase remains coherently bonded to the ductile matrix. This is possible due to non-random disorientations formed mostly in the Pitsch relation as well as the twinning of martensite.

The volume fraction of the secondary phase was identified in the direction perpendicular to the fracture trajectory. In order to match the microstructural analysis with a numerical model, the XFEM method allowing the macro-crack trajectory to be traced has been used. The plastic strain fields in front of the crack tip and along the crack edges were analysed. The kinetics of the phase transformation, assuming dependence of the rate of volume fraction of the secondary phase on the rate of accumulated plastic strain, as well as including the measured threshold strain and the relevant moderating function, was applied. The distribution of the volume fraction of the new phase was computed based on the distribution of the accumulated plastic strain, and compared with the microscopic measurements. Fairly good convergence between the microscopic observations and the numerical results was obtained. In particular, the distributions of the secondary phase in the directions parallel and normal to the crack trajectory were confirmed. Due to the rather high concentration of martensite observed at the crack tip (more than 70%), a strong recommendation to select materials with a limited phase transformation is expressed. In areas with a high martensite content, between the secondary phase grains, random boundaries arise, while the strong twin ones constitute a small fraction. In addition, ε martensite is often located along the boundaries. The identified microstructure is a medium of facilitated propagation of the macro-crack. Thus, safe performance of the structural elements at cryogenic temperatures requires a significant reduction of the phase transformation. The martensite content should be less than 20%. Then, it plays the role of the strengthening phase. Finally, the above results clearly show the significant complexity of fracture in two-phase continuum, accompanied by constant evolution of the proportion between the phases during the macro-crack propagation.

## Figures and Tables

**Table 1:** Chemical composition of 316L specimens.

Element	C	Si	Mn	P	S	Cr	Ni	N
**Content [%]**	≤0.030	1.00	2.00	0.045	0.015	18.200	11.500	-

**Table 2:** Volume fractions of key austenite orientations depending on temperature and distance from the specimen fracture surface.

Temperature	4.2 K		77 K		RT	
Distance from the fracture surface	4 mm	2 mm	4 mm	2 mm	4 mm	2 mm
The Brass orientation	8.1%	8.8%	9.5%	10.6%	9.8%	10.0%
The Goss orientation	6.9%	6.9%	7.5%	6.8%	8.4%	8.1%
The Cu orientation	4.2%	3.9%	5.9%	5.8%	6.4%	6.0%

**Table 3:** Parameters of the material model.

Material Constant	*E* [MPa]	*σ_0_* [MPa]	*C_R_* [MPa]
sample 1	220.000	880	7750
sample 2	210.000	880	7890

## Data Availability

Data is contained within the article.
